# ADAR1: a mast regulator of aging and immunity

**DOI:** 10.1038/s41392-022-01276-5

**Published:** 2023-01-04

**Authors:** Yigan Zhang, Jinyue Zhang, Yuanchao Xue

**Affiliations:** 1 Key Laboratory of RNA Biology, Institute of Biophysics, Chinese Academy of Sciences, 100101 Beijing, China; 2 University of Chinese Academy of Sciences, 100049 Beijing, China

**Keywords:** Epigenetics, Inflammation

Recently, *Nature* and *Nature Cell Biology* published five papers on the function and molecular mechanism of ADAR1 (adenosine deaminases acting on RNA) in aging, cancer, and autoimmune diseases.^1–5^ Among them, four papers published in *Nature* revealed that ADAR1 regulates autoimmune disease and cancer immunotherapy through canonical adenosine-to-inosine (A-to-I) RNA editing.^1–4^ Using a different approach, the paper in *Nature Cell Biology* discovered that ADAR1 could suppress senescence by regulating *p16* ^*INK4a*^ expression through an RNA editing independent non-canonical pathway.^5^

**Fig. 1:**
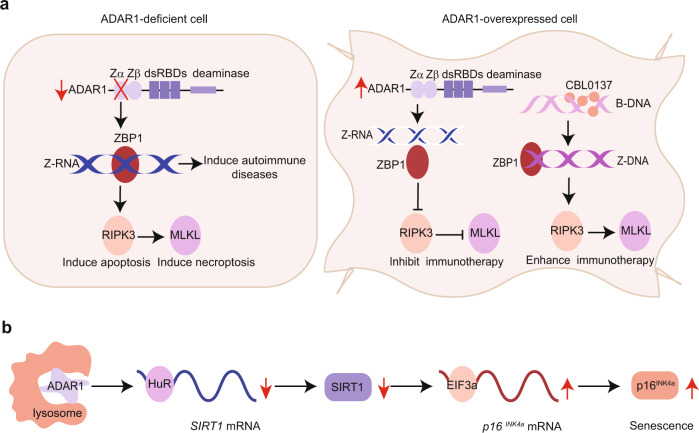
ADAR1 regulates immune response and senescence through A-to-I RNA editing dependent and independent pathways, respectively. **a** Mutation of the ADAR1 Zα domain or ADAR1 depletion causes deficiency in A-to-I RNA editing, leading to the accumulation of Z-RNA. The accumulated Z-RNA subsequently activates ZBP1 to drive autoimmune diseases by inducing necroptosis or apoptosis in ADAR1-deficient cells. In contrast, ADAR1 tends to be overexpressed in cancer. The elevated expression of ADAR1 will reduce Z-RNA accumulation through A-to-I RNA editing, thereby inhibiting ZBP1 activation and ZBP1-mediated programmed necrosis of cells. Considering the similarity between Z-DNA and Z-RNA in structure, Zhang et al. screened a small molecule, CBL0137, that can transform a large amount of B-DNA into Z-DNA for activating ZBP1 to enhance immunotherapy. **b** In aging cells, ADAR1 is downregulated by the lysosomal-mediated autophagy pathway, which decreases the association between HuR and *SIRT1* mRNA, leading to the significantly reduced stability of *SIRT1* mRNA. The reduced expression of SIRT1 will further promote the association of EIF3a with *p16*^*INK4a*^ mRNA to accelerate its translation. This A-to-I independent pathway appears to be a mast regulator of cell senescence through modulating *p16*^*INK4a*^ levels

The classical function of ADAR1 is to recognize double-stranded RNA formed by internal repetitive complementary sequences and catalyze the deamination of adenosine to inosine. Due to the inability of hypoxanthine to pair with uracil, the A-to-I editing tends to disrupt double-stranded RNA structures and thereby modulate RNA metabolism post-transcriptionally. Coinciding with its crucial role in RNA editing, researchers later found that dynamic changes in ADAR1 expression levels and protein activity could promote the progression of severe chronic diseases such as asthma, Crohn’s disease, and hepatitis B. These studies suggest that the homeostasis of ADAR1 is required for maintaining health and modulating its expression may be beneficial in treating various diseases. However, the underlying functional mechanisms of ADAR1 in disease progression remain unclear (Fig. 1).

On July 20, 2022, *Nature* published three papers on ADAR1 regulating abnormal immune activation through the canonical A-to-I RNA editing pathway.^1–3^ The study by Nicholas et al. demonstrated that ADAR1 negatively regulates the activity of ZBP1, and ZBP1 can promote autoimmune diseases in the absence of ADAR1 Zα domain function.^1^ However, knockout of ZBP1 rescued the phenotypic changes caused by ADAR1 Zα domain loss-of-function. Their study also showed that RIPK3 knockout phenotypes were similar to ZBP1 knockout phenotypes. However, simultaneous knockout of caspase-8 and RIPK3 or caspase-8 and MLKL aggravated the pathogenic effect of ADAR1 deletion. Richard et al. also demonstrated that ADAR1 could inhibit ZBP1-mediated apoptosis.^2^ Their studies showed that the Zα domain of ADAR1 can inhibit ZBP1 by recognizing dsRNA, restraining caspase-8-dependent apoptosis and MLKL-mediated necroptosis. Furthermore, ADAR1 can also reduce the expression level of dsRNA through A-to-I editing of the endogenous Alu element, thereby inhibiting the activation of ZBP1. Jiao et al. showed that ZBP1 is a key molecule that promotes the pathogenic type I interferon response in hemizygous mice with ADAR1 Zα domain mutations.^3^

In line with the aforementioned loss-of-function studies, Zhang et al. showed that overexpression of ADAR1 could inhibit ZBP1 activation through Z-RNA editing, thereby preventing ZBP1-mediated programmed necrosis of cells and tumor immune activation.^4^ Conversely, targeted inhibition of ADAR1 can improve the activity of ZBP1 and activate RIPK3-MLKL-dependent programmed necrosis to enhance the efficacy of tumor immunotherapy. In terms of clinical transformation, since the structure of Z-RNA and Z-DNA are very similar, Z-DNA can also interact with the Zα domain of ZBP1 to activate ZBP1. Therefore, Zhang et al. screened a small molecule compound CBL0137, which can strongly induce the conversion of B-DNA to Z-DNA and then activate ZBP1. Animal experiments showed that CBL0137 combined with an anti-PD-1 antibody could improve the efficacy of immunotherapy.

On July 18, 2022, *Nature Cell Biology* reported an interesting observation from Xue et al. who proposed a novel mechanism by which ADAR1 regulates gene expression independent of A-to-I editing.^5^ Xue et al. found that ADAR1 could promote the interaction between HuR and *SIRT1* mRNA, thereby increasing the stability of *SIRT1* mRNA. The elevated SIRT1 expression, in turn, suppresses the translation of *p16*^*INK4a*^ mRNA, thus inhibiting the occurrence of senescence. However, in aging cells, ADAR1 is degraded by lysosomal-mediated autophagy. Xue et al. found that the protein level of ADAR1 in the brain, ovary and other tissues of aging mice was significantly lower than that of young mice. Because the *ADAR1* mRNA expression level did not change significantly, this result indicates that the down-regulation of ADAR1 expression during aging mainly happened at the post-transcriptional level. Xue et al. also found that applying small molecule inhibitors targeting the lysosomal autophagy pathway in aging cells could inhibit the loss of ADAR1 expression due to aging. Overall, the study of Xue et al. revealed a novel mechanism of autophagy in promoting aging and indicated that the altered ADAR1 expression might be a biomarker of aging. Importantly, modulating ADAR1 expression levels may be potent in treating aging-related diseases (such as Parkinson’s disease, etc.).

These three articles jointly revealed that the loss of ADAR1 function can activate ZBP1-mediated autoimmune diseases and embryonic death, indicating that ADAR1 is negatively related to immune activation.^1–3^ The article of Zhang et al. also showed that ADAR1 is negatively correlated with immune activation, but they emphasized the mechanism of ADAR1 in tumor immunosuppression.^4^ The above four papers revealed the A-to-I editing mechanism of ADAR1, while Xue et al. showed that ADAR1 regulates aging in an A-to-I independent manner.^5^ These results have deepened the understanding of the function of ADAR1 and provided novel insights into the intervention and treatment of tumors, autoimmune diseases, and aging. As mentioned by Zhang et al., we currently lack specific small molecule inhibitors to ADAR1, limiting the development of therapeutic drugs to cure tumors and autoimmune diseases. At present, the Protein Data Bank has the crystal structure of the Zα domain and Zβ domain of ADAR1, and the Alphfold2 program has also predicted the complete structure of ADAR1 protein. We believe that virtual screening and molecular dynamics detection technology, such as Surface Plasmon Resonance and Microscale Thermophoresis, can be used to develop specific small molecule drugs that can directly inhibit the activity of ADAR1, and we believe this will promote the clinical translation of those recent exciting discoveries. In addition, because the primary target of ADAR1 is dsRNA and A-to-I editing can disrupt the dsRNA conformation, we speculate that global mapping of ADAR1-mediated RNA editing sites on dsRNAs in tumor cells and autoimmune disease samples may greatly promote our understanding of ADAR1’s molecular function. Although some methods seem feasible, developing novel approaches to detect ADAR1-mediated in situ RNA-RNA interactions is urgently needed to fill the remaining knowledge gaps.
